# 1164. Acquisition of Methicillin-Susceptible *Staphylococcus aureus* in the Neonatal Intensive Care Unit: Molecular Comparison of Relatedness of Isolates

**DOI:** 10.1093/ofid/ofab466.1357

**Published:** 2021-12-04

**Authors:** Dalia F Eid, Angela Gomez-Simmonds, Vitaliya Boyar, Anne-Catrin Uhlemann, Lorry G Rubin

**Affiliations:** 1 Cohen Children’s Medical Center/Northwell Health, Roslyn, New York; 2 Columbia University Irving Medical Center, New York, New York; 3 ccmc, northwell, New Hyde Park, New York; 4 Cohen Children’s Medical Center of NY, New Hyde Park, NY

## Abstract

**Background:**

In the NICU MSSA infections occur frequently and cause morbidity and mortality. Colonization is a risk factor for infection. Optimal infection prevention strategies await a more complete understanding of acquisition and transmission. To investigate possible transmission, we studied whether newly MSSA colonized infants share a strain with another contemporaneously colonized infant.

**Methods:**

This is a prospective observational study in a level IV NICU from April through November 2019. Infants had weekly MSSA nasal surveillance cultures. Isolates from newly MSSA colonized infants and other infants colonized with MSSA during the same/previous week were subjected to staphylococcal protein A (spa) typing; most pairs with a concordant (CC) spa type were analyzed by whole genome sequencing (WGS; Illumina MiSeq). Pairs of isolates with a CC spa type and < 25 single nucleotide polymorphism differences on WGS were considered closely related (CC pairs). A control group consisted of pairs of isolates from a newly colonized infant with one randomly chosen colonized infant with a discordant (DC) spa type during the same/previous week. The medical records were reviewed for staff member (SM) and room assignment. Fischer’s exact test was used to compare proportions.

**Results:**

Isolates from 60/68 consecutive newly MSSA colonized infants and 111/133 comparison infants were available for spa typing. Of these 60 infants, 23 (38 %) had a CC spa type with another infant colonized during the same/previous week. Of 18 isolate pairs from infants with a CC spa type that were subjected to WGS, 12 (67%) pairs of isolates were closely related. 7/12 (58 %) of CC pairs had a SM in common compared to 2/13 (15 %) in the DC pair groups, p=0.04. 2/12 (17 %) of CC pairs shared a room compared to 2/13 (15 %) pairs in the DC group, p=1.0.

**Conclusion:**

Among newly MSSA colonized infants at least 25% are colonized with an isolate closely related to that of another colonized infant indicating likely infant to infant transmission. WGS is more discriminatory than spa typing for MSSA. Given the lack of commonality of room assignment and the commonality of SM assignment, a possible role of healthcare personnel in MSSA transmission should be further investigated.

**Disclosures:**

**Anne-Catrin Uhlemann, MD, PhD**, **Merck** (Grant/Research Support)

